# The Surgery of the Pericardium

**Published:** 1882-01

**Authors:** 


### ORIGINAL TRANSLATIONS AND ABSTRACTS.

### THE SURGERY OF THE PERICARDIUM.

In an excellent paper (*Annals of Anatomy and Surgery*, December, 1881) Dr. John B. Roberts advocates aspiration, and if necessary drainage or injection of the pericardial sac with irritating fluids in order to excite adhesive inflammation of the walls of the sac. Dr. Roberts points out the indications for the operations as follows:

In all cases of pericardial effusion that present dangerous symptoms of heart failure, aspiration should be performed as soon as it is evident that medication is not lessening the embarrassment of the central organ of circulation. It is bad practice to delay the operation until exhaustion, pulmonary engorgement and degeneration of cardiac muscle render permanent relief impossible. In nearly all instances the tendency is to wait too long, instead of operating promptly and affording immediate relief. Clinical experience has abundantly shown that when the pericardial fluid is evacuated the dyspnœa, cyanosis, irregular pulse and other threatening symptoms are lessened, and usually at once. The time for operation depends less on the amount of fluid than would at first be supposed, because the sudden effusion of a moderate amount of serum will exert more pressure upon the heart than a much larger quantity poured out in so gradual a manner as to allow the pericardium to become stretched. Hence: aspirate in all cases of effusion, in which dangerous symptoms of heart embarrassment occur, as soon as medication fails, without regard to the supposed quantity of fluid.

If there coexists pleural effusion of considerable amount, the pleural sac should be aspirated first, because it is difficult to discriminate between respiratory distress due to pulmonary pressure and that resulting secondarily from interference with cardiac action. This rule applies to pleurisy of the right side as well as of the left.

When the amelioration of symptoms following pericardial aspiration is not permanent, because reaccumulation takes place, the operation should be repeated. It is better to vary the point of puncture lest, on account of adhesion of the layers of pericardium at the original point, the heart be wounded at the second tapping. Should repeated tapping be demanded, it might be considered proper, after the third operation, to inject some irritating fluid, such as tincture of iodine, into the sac, with the idea of producing adhesion of the two layers of the pericardium. Universal pericardial adhesion is found after cure by simple tapping, and injection has been done without preventing, or apparently interfering, with recovery. The fluid injected ought probably to be concentrated, as the object to be obtained is a pericarditis which will furnish plastic lymph instead of serum. Carbolic acid undiluted, as used in hydrocele, would be the proper agent, were it not for the possibility that its contact with the heart walls might induce cardiac spasm.

When aspiration has shown the pericarditis to be distinctly purulent, it is almost certain that repetition of the operation will be demanded. If a second tapping is required, the introduction of a carbolized drainage tube, after a free incision has been made, seems the most judicious kind of surgery. The cavity can be washed out daily with antiseptic solutions, and purulent accumulation, with its attendant dangers of pressure on the heart and septicæmia, avoided. Empyema is known to result most favorably when so treated, pericardial fistulæ seem no more dangerous than pleural fistulæ, and pus allowed free egress from serous cavities under antiseptic precautions, is much less disastrous in its effects than when partially removed by the repeated use of the aspirator.

The best point for aspiration of the pericardium is in the fifth inter-space just above the sixth rib, about 5 or 6 centimeters (2–2¼ inches) to the left of the median line of the sternum. In a child it should be nearer the sternum. This point is outside of the line of the internal mammary artery, is in a wide portion of the intercostal space, corresponds with the notch in the border of the left lung, is low enough to preclude wounding the auricle and high enough to avoid the diaphragm, and does not approach the point where a cartilaginous band usually joins the fifth and sixth cartilages. Both layers of the pleura will probably be pierced by the aspirating needle at this point, but this is not an important complication, and can only be avoided by going close to the sternum, which is objectionable on other grounds.

The aspirating trocar devised for the purpose, consists of a moderate sized aspirating needle, flattened at its upper extremity to give the surgeon a firm hold, within which slides a canula. The distal end of the canula, made flexible by a spiral, when thrust beyond the point of the needle curves downwards, and thus prevents the point of the puncturing instrument injuring the heart when the fluid is nearly evacuated. During penetration of the thoracic wall the canula is retracted so that the flexible end is contained within the needle. The perforation at the end of the canula allows fluid to escape as soon as the pericardial sac is entered. The canula is then thrust forwards until the sharp point of the needle is guarded. This movement brings a lateral fenestra in the canula opposite a similar opening in the needle, and thus provides a second orifice for the escape of fluid in case the terminal one becomes occluded. The external end of the canula has a square shoulder to prevent rotation within the needle, and terminates in an enlargement for attachment to the tube of the aspirator. The needle, or outer canula as it may be called, is marked on the surface to show the number of centimetres concealed in the tissues. If the inner canula is suspected to be clogged with lymph-shreds or pus, it can be withdrawn without disturbing the needle ; and the attachment of the pump may then be made to the latter as if it were an ordinary aspirating needle.

